# The Ethical Issues of Learning Analytics in Their Historical Context

**DOI:** 10.1007/978-981-15-4276-3_3

**Published:** 2020-05-15

**Authors:** Dai Griffiths

**Affiliations:** Research Institute for Innovation & Technology in Education (UNIR iTED), Universidad Internacional de La Rioja (UNIR), Logroño, La Rioja, Spain; The University of Bolton, Deane Road, Bolton, BL3 5AB UK

## Abstract

The ethical context of Learning Analytics is framed by two related processes. Firstly, the amount of personal data available to organisations has been transformed by the computerisation and the subsequent development of the Internet. Secondly, the methods and ethical assumptions of Operations Research have been extended into new areas. Learning Analytics can be conceptualised as the extension of Operations Research methods to educational institutions, in a process facilitated by technological and social changes in the early twenty-first century. It is argued that the ethical discourse has viewed Learning Analytics as a discrete field, and focused on its internal processes, at the expense of its connections with the wider social context. As a result, contradictions arise in the practice of research ethics, and a number of urgent issues are not given due consideration. These include the partial erosion of the consensus around the Nuremberg code; the use of ethical waivers for quality improvement; the coercive extraction of data; the use of analytics as an enabling technology for management; and the educational implications of the relationship between surveillance and trust.

## Ethics and Learning Analytics

As a first step in considering the ethical context in which Learning Analytics (LA) is carried out, it is necessary to discuss the declared purpose and modus operandi of the field. Writing at an early stage in the development of LA, Long and Siemens, two of the principal actors in the field, explained the rationale for the development of LA, arguing that research indicates that “data-driven decision-making improves organizational output and productivity”, and that education is falling behind other fields in this respect.

Higher education, a field that gathers an astonishing array of data about its “customers,” has traditionally been inefficient in its data use, …. Organizational processes—such as planning and resource allocation— often fail to utilize large amounts of data on effective learning practices, student profiles, and needed interventions. Something must change. For decades, calls have been made for reform in the efficiency and quality of higher education. Now, with the Internet, mobile technologies, and open education, these calls are gaining a new level of urgency. (Long & Siemens, 2011)

Long and Siemens (2011) go on to distinguish between LA, which is “exclusively on the learning process” (p.34) and ‘academic analytics’, which is “the application of business intelligence in education” to achieve what Siemens elsewhere referred to as “organisational efficiency” (Long and Siemens, 2011). Since then a great deal of work has been done to implement LA systems, which has been best documented by the Society for Learning Analytics Research (SOLAR), in its annual Learning Analytics and Knowledge (LAK) conference series, and in the Journal of Learning Analytics which SOLAR publishes. It soon became clear that this work raised ethical issues, and Gasevic, Dawson and Jovanovic (2016) comment that “questions related to privacy and ethics in connection to learning analytics have been an ongoing concern since the early days of learning analytics”), with major questions including protection of personal data and data sharing.

Slade and Prinsloo have taken a leading role in identifying the ethical challenges faced by the implementers of LA, and their papers provide a good picture of the issues which were of concern to the field. In an early paper they discussed a number of ethical issues and dilemmas faced by LA (Slade & Prinsloo, 2013), identifying and exploring three broad overlapping categories of ethical issues:

1. The location and interpretation of data
2. Informed consent, privacy and the de-identification of data
3. The management, classification and storage of data.

An influential checklist of ethical concerns proposed by Draschler and Greller (2016) serves as an elaboration of some aspects of the three categories. A fourth important aspect, the obligation to act on knowledge, was identified in Prinsloo and Slade (2017). A recent report by Slade and Tait indicates the current concerns in the field:

…Core Issues that are important on a global basis for the use and development of Learning Analytics in ethics-informed ways:

- Transparency
- Data ownership and control
- Accessibility of data
- Validity and reliability of data
- Institutional responsibility and obligation to act
- Communications
- Cultural values
- Inclusion
- Consent
- Student agency and responsibility
  (Slade & Tait, 2019, p.1)

Readers are directed to a recent paper by Kitto and Knight (2019) which cogently analyzes these ethical initiatives and discusses the practical implications for building LA systems.

The extent of the discussion of ethics in the LA community is testament to the importance which it has been given, and the desire of the participants to act ethically, and to create systems which will not be rejected by stakeholders. Important issues have been identified, the possible courses of action analyzed in depth, and recommendations are made. The designers of LA systems are tasked with providing particular functionality, and they need guidance on how to go about this in an ethically defensible way, and without alienating stakeholders. These are important matters, which have been addressed in the literature which I cite above. In this chapter, however, I am concerned less with the internal workings of the field of LA, and more with the ethical implications of the connections between LA and the wider social context in which it is situated.

In response to the ethical considerations which I have described above, a number of institutions have established codes of practice for LA, to make explicit what will and will not be done with student data, and the roles of those involved. Such policies include, in the UK, Jisc (2015) and The Open University (2014c); in Canada, The University of British Columbia (undated); and in Australia, Charles Stuart University (2015). These codes of practice have the great merit of making explicit the basis for LA design, and they are the results of many person-years of reflection by experts in the field. Nevertheless, contradictions can arise between ethical practices in LA and those prevailing in academic research. The Open University (OU) of the UK provides a good example, because of the exemplary clarity of its policies on research ethics and on LA. The OU FAQs on LA inform students that in order to have “a complete dataset” available to the University “…it is not possible, at present, to have your data excluded” (The Open University, 2014b). At the same time, the OU’s Ethics Principles for Research Involving Human Participants assert that “Except in exceptional circumstances, where the nature of the research design requires it, no research shall be conducted without the opt-in valid consent of participants.” and that “Participants … have a right to withdraw their consent at any time up to a specified date” (The Open University, 2014a). LA at the OU is intended to “identify interventions which aim to support students in achieving their study goals” (The Open University, 2014c), an aim which has traditionally been addressed by means of academic educational research. From these documents, it inescapably appears that an OU PhD student who is researching into teaching and learning outside the OU would need to obtain ethical approval and consent from participants before gathering any data, while the student’s parent organisation carries out the same research on its own students without any such constraint. All three OU documents that I have cited are dated 2014, and so this contradiction is not a case of outdated policies failing to keep track of recent developments.

The OU is by no means alone in viewing very similar research activities through quite different ethical lenses. To take another example, the Charles Sturt University Learning Analytics Code of Practice states that data is collected without consent:

Data is collected from learning and teaching systems, retained and utilised for the purposes of enhancing learning and teaching by: (…)

- Contributing to research and scholarship in learning and teaching (Charles Sturt University, 2015)

At the same time the University ascribes to the National Statement on Ethical Conduct in Human Research, which states that

Consent to participate in research must be voluntary and based on sufficient information and adequate understanding of both the proposed research and the implications of participation in it. (NHMRC, 2018)

I have actively promoted LA for a number of years, and it is not my intention here to proclaim *mea culpa,* or to condemn my colleagues for failing to abide by codes of practice for research with human participants. Rather I seek to achieve some understanding of the shifting context which has caused well-intentioned and conscientious people to find themselves in this tangle, and to provide a way of conceiving of the forces which are shaping the future of education. To do this, we need to examine the historical context within which LA has developed.

## Two Traditions of Research Ethics

The academic community working on LA has defined itself as a distinct field of study. This makes it possible to maintain a scholarly community and discourse, but tends to disguise the degree to which the ethical issues of LA are not specific to the field, but rather are symptomatic of wider changes in which networked data is used to mediate and mould human relationships. To understand these changes, and their ethical implications, it is necessary to look back in the past, well beyond the development of the Internet. Knowingly or otherwise, practitioners of LA, academics and ethical review bodies have all situated themselves within ethical traditions which date back to the Second World War when framing the use of data in understanding and predicting the activities and performance of organisations and individuals.

Following the Nuremberg trials of those involved in Nazi atrocities during the Second World War, the Nuremberg Code (U.S. Government, 1949) was established to ensure that research would never again establish an abusive or exploitative relationship with its subjects. According to Shuster (1997), the Code has not been officially adopted by any nation or major medical association, but “Nonetheless, its influence on global human-rights law and medical ethics has been profound.” Of particular relevance for this chapter is article 1 of the Code:

The voluntary consent of the human subject is absolutely essential …the person involved should … be able to exercise free power of choice, there should be made known to him the nature, duration, and purpose of the experiment; the method and means by which it is to be conducted …. (U.S. Government, 1949)

The Code was originally conceived in response to horrific Nazi-sponsored medical research and it was in medicine that it was first applied. However, the application of the Code was soon extended to all research involving human subjects. This is reflected in all major policies and codes on ethics in the social sciences, including the influential Common Rule in the U.S.A. (U.S. Government, 2017). For example, the International Sociological Association (2001) code of ethics states that “The consent of research subjects and informants should be obtained in advance”, while the British Educational Research Association Ethical Guidelines for Educational Research affirm that “It is normally expected that participants’ voluntary informed consent to be involved in the study will be obtained at the start of the study” and stipulate that “It should be made clear to participants that they can withdraw at any point without needing to provide an explanation” (British Educational Research Association, 2018, p.9).

Also with its origins in the Second World War, a quite different ethical tradition has developed. During the War, researchers were employed to optimise the operation of the military and other essential government services. This was particularly the case in the application of new technologies, such as radar, which required the development of new collaborative sociotechnical processes to achieve optimal performance. Rau (2005) describes how work was carried out

…to develop new filtering methods, study the effects of the location of stations on radar performance and discover why some aircraft slipped through the radar network undetected. Rowe referred to this work as ‘operational research’ to contrast it with the ‘developmental research’ going on in the laboratories and workshops: in OR the work took place ‘on site’.

‘Operational’, or more frequently ‘Operations’ Research (OR) continued in peacetime, with investigations being carried out in businesses, industrial organisations and the state sector, with the aim of establishing effective processes and management strategies. As Rau continues, “the subsequent influence that these wartime foundations had on OR are hard to overestimate”. In the 1960s computer models started to be used extensively to support OR, with the development of Decision Support Systems (see Ferguson and Jones (1969) for an early example). ‘Business Intelligence’ can also be seen as a manifestation of OR, while Stafford Beer (1967) viewed the whole of Management Science as “the business use of operations research”. It is therefore clear that we are looking at a continuous and influential tradition of research in these fields leading back (at least) to the Second World War, whatever disagreements there may be about how to name the parts and the whole.

Many in academia will, I suspect, be unfamiliar with the OR tradition and its related fields, and perhaps even doubt that its activities should be classified as ‘research’. However, an early definition by Pocock describes OR as follows:

Operations Research is a scientific methodology analytical, experimental, quantitative which, by assessing the overall implications of various alternative courses of action in a management system provides an improved basis for management decisions. (Pocock, 1956)

Thus, OR applies scientific methodologies to understand the world, and as such it seems undeniably to constitute ‘research’. If it is unfamiliar in academia, outside areas such as management science and information systems, this may be because its objects of study have not overlapped with those of academic researchers. Once academics accept OR as ‘research’ in the full sense of the word, often working with human respondents, they may assume without further reflection that ethical codes of practice drawn from the Nuremberg Code will be applicable to it, but this has not been the case at any point in the history of OR. The lack of ethical scrutiny in OR is not simply a matter of ignorance or evasion. An athlete can keep records of their own performance, and use this data in optimising their diet and exercise regime, without the requirement for any ethical approval. Similarly, the organisation can be seen as a metaphorical (and, in many cases, legal) person that owns its own data, and has the freedom to do as it wishes with it to optimise its own performance. As Picavet comments “in operational research, efficiency is not usually viewed as something which conflicts with ethics. Quite simply, it does not refer to the same category of problems.” (Picavet, 2009, p. 1122). Moreover, the methods and processes which result from this research may bring significant competitive advantage to the organisation. It is therefore not surprising that opening up this research to ethical scrutiny is not only seen as unnecessary, but is also actively unwelcome, as it threatens the secrecy required to protect competitive advantage. There has been ongoing discussion of ethics within the OR community over a number of years, see, for example Le Menestrel and van Wassenhove (2009). However, where ethical codes for OR exist, they make no mention of the key dispensations of the Nuremberg Code, in particular those of informed consent and of a right to withdraw. This is the case, for example in the ethical principles of the Operational Research Society (2019).

I have argued for the continuity of thinking on research ethics since the Second World War, but this thinking has also evolved over time, principally as a result of the impact of technological change, as I now discuss.

## The Impact of Technology on Research Ethics

The massive expansion of the availability of data on human interactions generated by the Internet has had a profound impact on assumptions about the ethics of gathering data. To illustrate the nature of these changes it is sufficient to look back at the concerns about computing in the years before the emergence of the Internet. Culman and Smith (1995) describe the furore that surrounded Lotus Marketplace. Launched in 1990, this was a CD-ROM for sale at $695 containing information about 120 million Americans, including name, address, age, gender, marital status, household income, and lifestyle and purchasing propensities. Software was included to facilitate creation of mailing lists that targeted prospective customers. 30,000 letter writers and callers contacted Lotus, complaining that the product was a violation of privacy. The CEO of Lotus concluded that the company “would be ill-served by a prolonged battle over consumer privacy”, and the product was cancelled in January 1991. Despite the reticence of Lotus, that battle has since taken place, and has resulted in an unqualified victory for commercial interests over personal privacy. Today the scale of data held by Lotus Marketplace, and the threat of increased junk mail which it presented, both seem insignificant. The supermarkets where we shop (Rowley, 2007) and the websites which we visit (Evans, 2009) hold and exchange vastly more detailed data about us than Lotus could have dreamed of. The revelations of Snowdon (Greenwald, 2014) have shown that governments have secretly developed vast infrastructures to monitor and analyze the communications of individuals. Cambridge Analytica, the firm accused of harvesting millions of people’s data from Facebook, “said publicly that it held up to 5000 data points on each of over 230 million American voters.” (Mr. Justice Norris, 2019). While this case ended in litigation, no effective opposition has emerged to the gathering of data on a massive scale by private companies, and its use in marketing, advertising and politics. Similarly, within the field of LA the InBloom analytics initiative was closed following a public reaction to intrusive data gathering (Bulger, McCormick & Pitcan, 2017), but this has not slowed the growth of the field.

Zuboff (2019) argues that Google has taken a leading role in these developments and provides a detailed analysis of the strategy established by Google Chief Economist Hal Varian to leverage the data gathered by the company. By 2014 Varian was confident enough to assert that these new procedures with private and personal data had become accepted practice rather than a matter for discussion:

‘There is no putting the genie back in the bottle … Everyone will expect to be tracked and monitored, since the advantages, in terms of convenience, safety, and services, will be so great … continuous monitoring will be the norm (Varian, H, quoted in PEW Research (2014)).

Zuboff herself has painted a less optimistic view of this outcome:

…our lives are unilaterally rendered as data, expropriated, and repurposed in new forms of social control, all of it in the service of others’ interests and in the absence of our awareness or means of combat. (Zuboff, 2019, p.54)

Zuboff (2019) identifies as ‘surveillance capitalism’ the methods which have driven the expansion of the analysis of personal data since the emergence of the Internet. Its principal actors are commercial organisations and security agencies, its workings are shrouded in secrecy, and its purpose is to obtain personal or organisational advantage. LA, in contrast, is a self-declared community of academics working towards an explicit goal of improving education, and sharing their methods and insights in public conferences. This contrast means that the emergence of LA cannot be explained as simply a manifestation of surveillance capitalism. Nevertheless, LA shares some of the methods and assumptions of surveillance capitalism, and these carry with them assumptions about data gathering and use which may shed light on the ethical tangle which LA finds itself in. These assumptions were well summarised by Bill Schmarzo, chief technical officer of EMC Global Services: “I’m a hoarder, I want it all. And even if I don’t yet know how I’ll use that data, I want it … My data science team might find a use for it” (Bertolucci, 2014). Informed consent for a specified purpose, is clearly not compatible with such a strategy.

The changes which have taken place in the practice of data collection in society have had a substantial impact on the methodology of the social sciences, to the extent that it has been seen as constituting a ‘watershed moment’ (McFarland, Lewis and Goldberg, 2015). The increasing influence of ‘big data’ data-gathering strategies is blurring the line between OR and academic social science in two ways.

Firstly, OR is being applied in fields which were once the preserve of academic research. Until the emergence of the Internet, the data available to OR researchers was largely limited to that produced in-house by commercial and governmental organisations, such as production process data and internal communications. This led to a well-demarcated area of research, which studied the internal processes of organisations, and seldom clashed with academic research investigating wider social phenomena. Today, however, huge quantities of data are available, generated by clients and stakeholders’ engagement with computer systems, which enable organisations to address a much wider range of OR questions, many of which are situated beyond the confines of the organisation per se. For example, retailers would in the past have conducted operations research on their organisation and communications, but would usually have relied on social scientists to analyze the social context in which they were operating. Now, however, an advertisement for analytics roles at TESCO says that successful applicants will “help the business to really understand our customers and suppliers” (TESCO, 2019). Similarly, ‘predictive policing’ practices are encroaching on the domain of academic criminology, using data analytics “to identify liktargets for police intervention and prevent crime or solve past crimes by making statistical predictions” (Perry, 2013 p. xiii), combining, for example, GPS tracking, license plate readers, and geographic profiling tools (Perry, 2013; Table 10.1007/978-981-15-4276-3_5).

Secondly, academic social science has adopted OR methods. According to Gary King, Director of the Harvard Institute for Quantitative Social Science, “Businesses now possess more social-science data than academics do” (Shaw, 2014). In the face of this shift in power, many social scientists appear to have accepted that a change in research practices is inevitable, with concomitant ethical implications which have yet to be spelt out. For example, the Social Science Research Council has entered into the Social Science One collaboration with Facebook. This development has been welcomed by some social scientists, for example Puschmann (2019), while the complaints of the European Advisory Committee for Science One (2019) are that “Facebook has still not provided academics with anything approaching adequate data access”. In contrast Leetaru (2019) argues that Social Science One will make the personal and intimate data of two billion Facebook users available for data mining by researchers, with little information available about the details of aggregation, privacy or how the results of the research might be used in intervening in society. In the context of developments such as this, it is unsurprising that LA practitioners are unwilling to commit to the constraints which would be placed on their relatively small scale studies by compliance with policies on research ethics with human participants.

The argument made here is that within the wider context of the influence of data analytics on the social sciences, the development of LA can be best understood as the extension of the OR research tradition to the education sector. Furthermore, the contradictions identified above between ethical policies for academic research with human subjects, and ethical guidelines for the practice of LA, correspond closely to the tension between the ethical traditions of OR and academic research. I propose that the resolution of these ethical contradictions can only be achieved through an understanding of the implications of these changes for educational systems, institutions and the people who work and study in them, and then taking decisions in the light of that understanding. In the remainder of this chapter, I will consider some of the issues which will need to be addressed in order to achieve such an understanding.

## Ethical Issues Raised by the Extension of Operations Research to Education

### The Erosion of the Nuremberg Tradition

The multiple issues and challenges of the ethics of LA may be summarised in this quandary: is LA academic research (as it appears to be from its objects of study) or is it OR (as it appears to be from its institutional purpose)?

This quandary is easy to name, but hard to resolve. A resolution in favour of either alternative implies consequences that are unacceptable to many actors. If LA is research, and is governed by the Nuremberg code, then education institutions will be required to seek consent from students and teachers for any analytical activity, in advance, and to specify the purpose of the data gathering. As we have seen above, this flies in the face of universal education practice in maintaining records of student attendance and achievement, and would rule out many of the activities of LA. It would also have significant consequences for commercial suppliers of services to education. On the other hand, if, as I have argued, LA is most usefully conceptualised as the extension of OR to the institution, then there is a prospect that the entire ethical framework for educational research could become irrelevant. There are two ways in which this may take place. Firstly, in the medium term the availability of data on the systems of institutions and their commercial partners, and the ease of access and ethical approval through LA, will inevitably be an attraction to educational researchers, and a cause for cynicism about research ethics procedures. It is hard to see how this could not undermine the authority of research ethics procedures.

Secondly, educational research could simply move out of the academic sector. To take an example, researchers from Pearson presented a paper at the American Educational Research Association Annual Meeting (Belenky, 2018) which described a randomised control trial in different messages that were embedded in the MyLab Programming application, in order to see which would lead students to attempt and complete more problems. Five thousand students were unknowingly involved in the experiment. According to Herold (2018) “the research prompted a fierce debate over issues of ethics, privacy and consent” leading to a fall in stock value. Pearson then described the experiment as a “relatively minor product update” (Herold, 2018) No related research has been published by Pearson, but institutions who use their products cannot know if the company is no longer carrying out such work, or if such analytics work is simply not being reported. The same is true, of course, for other companies. The question for educational institutions is whether it is ethical to make use of educational services which are not sufficiently transparent in their use of data, when doing so means that they cannot ensure that their institutional codes of practice for both research ethics and LA are being respected.

The issues raised by the Pearson research are a particular case of a wider process. As discussed by Zuboff (2019 p.299–305) the issue was brought to prominence by a paper in Nature by University of California and Facebook researchers leveraging company data, entitled “A 61-million-person experiment in social influence and political mobilization” (Bond et al., 2012). The editor of Nature said in an interview that

I was concerned … until I queried the authors and they said their local institutional review board had approved it—and apparently on the grounds that Facebook apparently manipulates people’s News Feeds all the time. (Lafrance, 2019)

The editor also explained that the review board had approved the study because it used a ‘pre-existing dataset’. Professor Chris Chambers summarised the concerns of many that

the Facebook study paints a dystopian future in which academic researchers escape ethical restriction by teaming up with private companies to test increasingly dangerous or harmful interventions. (Chambers, 2014)

If, as I have argued, LA wholly or partly represents the extension of OR to the Education sector, then a primary ethical concern for the sector should be the disruption which this extension is causing to research ethics within education, and the likely implications of its future impact on education and educational research. A failure to conduct this discussion would mean that, despite all the good intentions and reflection in the LA community, the ethics of LA would be those of a power grab: ‘we grant ourselves an ethical exemption because we can, and let the social consequences fall as they will’.

### Ethical Waivers and Exemptions

The medical field has been confronted with very similar ethical issues to those raised by LA, but for rather longer because until recently much more data was generated in the health sector than in education. In many jurisdictions, including the United States, the same problem that we have identified in LA arises, i.e. deciding whether an investigation qualifies as ‘research’ (which is governed by ethical review boards) or as ‘quality improvement’ OR (which has a waiver or exemption from ethical review), as discussed by Goldstein et al. (2018). Taylor et al. (2010) describe how in the 1990s “questions were raised as to whether quality improvement initiatives ought to be considered human subject research and reviewed and regulated as such”, and they provide a number of examples.

These questions remain open, as McLennan writes:

The ethical oversight system in Switzerland currently places a higher standard of ethical oversight on “research” in comparison with “quality control” activities using the same quality data. However, these activities cannot often be reliably differentiated from each other and the inconsistent ethical oversight of these activities needs to be reconsidered. (McLennan et al., 2018)

Here we have a more mature field which is wrestling with the same issues as LA, and it is surely worth examining the parallels to see what can be usefully learned, but very little connection has been made between LA and medical ethics. In particular, the concept of an ethical waiver or exemption for quality improvement maps well onto the contradiction between LA and academic research in education. From this perspective, it is not sufficient for LA to publish codes of practice. The ethical challenge for LA is to discuss in one framework both the codes governing research with human subjects and those governing LA, and to justify the exemptions which are granted to LA. This discussion may result in desirable changes to the governance of research with human subjects, as well as adjustments to LA. It will have to take into consideration wider changes in society regarding the use of data, and so will have social and political dimensions. It therefore cannot take place only in the LA academic community within which most of the ethical discussion of LA has so far taken place.

### Coercive Extraction of Data

Citizens in many countries share data in many aspects of their lives. Every time they drive a car their license plate may be captured, when they walk in a public space their face may be automatically processed. Every time they send an email, actors such as GCHQ and Google monitor its content. Unless they take elaborate precautions, many websites that they visit will share their record of interactions with an advertising network such as Google’s Adsense. The citizen has little choice but to acquiesce in this data collection, because their daily lives depend on carrying out these activities.

School-age education is compulsory around the world, and higher education is, increasingly, also a requirement for life in a developed economy. In an environment where all educational institutions monitor the data generated by all students, citizens and children are confronted by the coercive analysis of data which goes well beyond the traditional data generated in the course of educational administration. It is perhaps in recognition of this that Charles Sturt University (2015) explicitly excludes the use in LA of data from email and other private online communication. Nevertheless, while educational institutions refuse to exclude students from datasets (for example The Open University, 2014b), LA codes of practice cannot be seen only as the individual choice of an institution, but rather also as symptomatic of a society-wide coercive extraction of data from its citizens, for good or ill. An analysis of the ethics of LA that aspires to go beyond the comparison of implementation approaches should recognise that the field is part of this wider trend, and take a position on it.

### Learning Analytics Entwined with Governance

While wanting to avoid suggesting that there was a golden age in which lecturers and students were aware of, and able to contribute to, institutional management processes, there is no doubt that Shattock is correct to assert that in the UK

from 2000 to 2016, the policy turmoil that accompanied the increasing marketisation of the higher education system and the introduction of a league table culture has led to the growth of powerful vice-chancellor-led executive teams, which have transformed governance practice and decision-making in many universities. (Shattock, 2017)

This concentration of power is also to be seen in many institutions across Europe, as described by Paradeise and Thoenig (2013), and is in many respects a global phenomenon driven by the New Public Management movement (see Lapsley, 2009). LA represents a potential or actual source of data to feed into the Key Performance Indicators and other methods which are used by managers to control the institutions which they are running. Whether or not learners and teachers are keen to use an LA intervention, they may nevertheless find that they have to do so if they are to maintain their studies or jobs. A corollary of this argument, if it is accepted, is that ‘learning’ and ‘academic’ analytics (see Long & Siemans, 2011) cannot be kept separate for ethical purposes. Education institutions compete on the basis of their effectiveness in teaching, and an essential business target is for them to meet the key performance indicators in teaching and learning set out for them by government agencies. Similarly, management decisions about teaching contracts and pay are influenced by teaching achievement.

As I have argued elsewhere (Griffiths, 2017), LA generates models of the institution and its operation. These models are conceived from particular perspectives and for particular purposes, generally by or for people in a management role, in part because it is managers who control budgets and who have access to data. These models then become an active element in the management of the institution and of the activities of lecturers and students, and can change relationships in the institution. Brans and Gallo offer two ways of viewing the ethics of OR practice which can be applied to this situation. One perspective is that of “those whose view on ethics is mainly internal, i.e. those who focus on the relation between OR/MS professionals and clients and on the way the modelling work is carried on” (Brans & Gallo, 2007), emphasising technical correctness and honesty. Within LA, such correctness would include respecting the relevant codes of practice and regulations covering the management of data. Brans and Gallo identify others for whom ethical professional behaviour “means taking always into account the effects on society and nature of the decisions derived from their analyses and models” (ibid). From this perspective a person concerned with the ethics of an LA implementation needs to look at the systemic impact of LA on the institution as a whole, and on all of the people who will be directly or indirectly affected by it. Given the complex intertwining of LA with systemic change in education, it seems incumbent to pay attention to these wider consequences of model making in LA, both positive and negative, in assessing the ethics of LA implementations.

### Surveillance, Trust and Learning

Long and Siemens describe how Learning analytics can assist all stakeholders to penetrate “the fog that has settled over much of higher education” (Long & Siemens, 2011, p. 40). Increasing visibility is generally understood as a good thing, but could it also be that the fog can sometimes be an essential enabler of education? Fried argued that

There can be no trust where there is no possibility of error. More specifically, man cannot know that he is trusted unless he has a right to act without constant surveillance so that he knows he can betray the trust. Privacy confers that essential right. (Fried, 1970, p.56)

Trust is not the same as a lack of surveillance, but surveillance acts to reduce trust. It is trust shown towards students that enables them to demonstrate autonomy and initiative, and to learn from their own mistakes. It can therefore be argued that while dispelling the fog in education may have befits, and it also changes the behaviour of students. Writing about the educational application of online forums before the emergence of LA, Dawson (2006) noted that “behaviour is altered when students are aware of surveillance techniques” and that “attention must be given to the manner in which online discussion forums efficiently construct new subjects that are both ‘productive’ and ‘docile’”.

I describe as ‘cognitive engineering’ this use of technology to construct a productive and docile subject who learns what is prescribed. It is the fulfilment of Skinner’s dream of a teaching machine (Skinner, 1958). To the extent that we can know and specify what is best for others to learn, and how they should learn it (and this can certainly be argued in some situations), then this approach may be justified, indeed perhaps ethically obligatory. However, the application of such an approach as a technocratic imperative, and the lack of trust which that would bring with it, would not only clash with political and ideological ideas of personal freedom, but also fly in the face of the requirements for education as they have been set out in the twenty-first century. For example, the key competences set out by the European Commission in the New Skills Agenda for Europe (Kraatz, 2017) include learning to learn, social and civic competences and a sense of initiative and entrepreneurship. It is hard to see how such reflective communication skills and intellectual autonomy can be developed if error is not allowed or, indeed, encouraged.

LA is not incompatible with trust, but trust raises ethical questions for the design and implementation of LA. To what degree should the fog of education be dispersed in order to monitored and optimise students’ behaviour? To what extent is a particular LA implementation a constraint on students’ personal development and autonomy? To what degree are privacy and trust (of students by lecturers, and of lecturers by managers), necessary in order for them to develop as autonomous learners and human beings? To what extent does a cognitive engineering approach imply an abandonment of teachers’ responsibility for their learners? These are practical questions for LA, and they all have significant ethical implications. The answers will be as complex and as socially situated as the arguments around the pedagogy which LA seeks to support.

## Concluding Remarks

Throughout this paper, I have argued that the discourse of the ethics of LA should not be only that of a community of educationalists and technologists reflecting on its own practice within a discrete field with its own ethical imperatives. It is also necessary to view LA as a manifestation in the education sector of wider trends which are transforming society, and to theorise where these trends have come from, where they are going to and what the place of LA is within them.

I have made reference to the extensive and valuable work has been done to articulate codes of practice and to define the ethical conduct of LA. My purpose has not been to critique that work, but rather to place it in context, to provide a diagnosis of the causes of the ethical tensions which it is wrestling with, and to identify wider ethical issues which, in my view, are raised by LA.

Technology has enabled an on-going transformation of educational relationships, and continues to do so. LA is situated at the point of contact between technological developments in society as a whole, and the particular practices of education, and so offers an opportunity to inquire into the ethical dimensions of that emerging relationship. With this in mind, I hope to have shown that the ethics of LA concerns not only the self-contained act of data collection, or the details of data governance, but also more complex and relative issues of who is doing what for whom, how and with what ends. This requires an engagement with pedagogy, politics and ideology which has so far been more conspicuous by its absence than its salience in the discourse around the ethics of LA.

Socrates said that when we speak of ethics “what we are talking about is how one should live.” (Williams, 2011). As the second decade of the twenty-first century reaches its close, there is puzzlement on all sides about why we live as we do, and how we should respond. How did xenophobic nationalism re-emerge in democracies? How did we let the ocean become filled with plastic? Why are we unable to do anything about climate change? Why do we work more hours every year? Why were we so unprepared for the coronavirus? The answer to these questions is, I suspect, that our attention has been misdirected towards problems of marginal importance, while unnoticed processes have been radically transforming our way of life. If we want to understand the ethics of “how one should live” with data analytics in education, then we need to ask if our attention is well directed, or if, as I suspect, we are failing to examine links between our use of technology and wider processes that are transforming the way that students and teachers live their lives. Such a failure would risk the emergence of unintended consequences which would horrify many enthusiasts for the powerful and potentially beneficial technology of LA.
